# Australian women's use of complementary and alternative medicines to enhance fertility: exploring the experiences of women and practitioners

**DOI:** 10.1186/1472-6882-9-52

**Published:** 2009-12-15

**Authors:** Jo-Anne Rayner, Helen L McLachlan, Della A Forster, Rhian Cramer

**Affiliations:** 1 Mother and Child Health Research, La Trobe University, 324-328 Little Lonsdale St, Melbourne, Victoria 3000, Australia; 2 Division of Nursing and Midwifery, La Trobe University, Bundoora, Victoria 3086, Australia; 3 Royal Women's Hospital, Carlton, Victoria 3053, Australia

## Abstract

**Background:**

Studies exploring the use of complementary and alternative medicine (CAM) to enhance fertility are limited. While Australian trends indicate that women are using CAM during pregnancy, little is known about women's use of CAM for fertility enhancement. With the rising age of women at first birth, couples are increasingly seeking assisted reproductive technologies (ART) to achieve parenthood. It is likely that CAM use for fertility enhancement will also increase, however this is not known. This paper reports on an exploratory study of women's use of CAM for fertility enhancement.

**Methods:**

Three focus groups were conducted in Melbourne, Australia in 2007; two with women who used CAM to enhance their fertility and one with CAM practitioners. Participants were recruited from five metropolitan Melbourne CAM practices that specialise in women's health. Women were asked to discuss their views and experiences of both CAM and ART, and practitioners were asked about their perceptions of why women consult them for fertility enhancement. Groups were digitally recorded (audio) and transcribed verbatim. The data were analysed thematically.

**Results:**

Focus groups included eight CAM practitioners and seven women. Practitioners reported increasing numbers of women consulting them for fertility enhancement whilst also using ART. Women combined CAM with ART to maintain wellbeing and assist with fertility enhancement. Global themes emerging from the women's focus groups were: women being willing to 'try anything' to achieve a pregnancy; women's negative experiences of ART and a reluctance to inform their medical specialist of their CAM use; and conversely, women's experiences with CAM being affirming and empowering.

**Conclusions:**

The women in our study used CAM to optimise their chances of achieving a pregnancy. Emerging themes suggest the positive relationships achieved with CAM practitioners are not always attained with orthodox medical providers. Women's views and experiences need to be considered in the provision of fertility services, and strategies developed to enhance communication between women, medical practitioners and CAM practitioners. Further research is needed to investigate the extent of CAM use for fertility enhancement in Australia, and to explore the efficacy and safety of CAM use to enhance fertility, in isolation or with ART.

## Background

Studies exploring women's use of complementary and alternative medicines (CAM) to enhance fertility are limited despite increasing evidence of CAM use during pregnancy [1-8]. CAM is typically described as outside orthodox biomedicine and includes a broad spectrum of modalities used to promote health and wellbeing and/or treat illness [9].

There has been a rise in the use of CAM as a health care option in recent years in Australia and overseas [10-15]. In 2004, Australians spent AUD$85 million on consultations with naturopaths and herbalists, excluding the costs of medicines [16], and in the last quarter to June 2007 over three million private health insurance reimbursements were made for CAM services alone [17]. In 2007 over 60% of Australians reported using CAM; 44% visited a CAM practitioner [18] and self-prescribed CAM was obtained from multiple sources included pharmacies (51%), supermarkets (33%) and health food shops (32%) [19]. Similar rates of CAM use are reported in the United Kingdom (UK) [15]; the United States (US) [20]; Germany [21]; Switzerland [22]; and Japan [23]. In 2007 in the US, out-of-pocket expenditure on CAM was US$33.9 billion and there were 354 million visits to CAM practitioners [24]. The Australian [18,25] and international [13,15,22,23,26] literature shows CAM users are more likely to be women, who are well-educated, employed on higher-than-average incomes, with private health insurance.

Reasons advanced to explain the increased use of CAM include: dissatisfaction with or poor outcomes associated with orthodox medicine [27,28]; a need for more control in healthcare decisions [22,29-34]; treatment of chronic illnesses [20,29,30,32]; the perceived technological or impersonal nature of orthodox medicine versus the perceived naturalness of CAM [33,35]; and the personalised nature of the interaction with CAM practitioners, coupled with the use of individually tailored interventions [33,36,37]. CAM are commonly used in conjunction with orthodox medical treatments, however information about CAM use is generally not provided to nor sought by doctors [10,18,23,35].

Infertility (defined as the failure to conceive after 12 months of regular unprotected sexual intercourse) affects 15 to 18% of Australian couples [38], and an increasing number of couples seek assisted reproductive technologies (ART) to achieve parenthood [39]. A large contributor to this is the rising age of women at first birth [40]. In 2005, 20% of Australian women giving birth were ≥ 35 years and 3% of all Australian births involved ART [17].

In Australia since 1990, ART services have been reimbursed through Medicare (the national universal healthcare scheme) and associated drug therapies are funded under the National Pharmaceutical Benefits Scheme. Since November 2000 there has been no restriction on the number of cycles or services that can be used by a couple [41]. The Australian government meets 80% of costs for ART services provided out of hospital once a set annual threshold is reached. While there are Medicare and private health insurance rebates, couples are considerably out-of-pocket [42].

Like Australia, many other developed countries provide public funding for ART although this varies significantly [43]. However, many countries apply restrictions to publicly funded ART such as limiting the number of cycles, ineligibility for women over 40 years of age and waiting lists for treatment [41,44]. In the United States the costs of infertility treatment are borne by the couple [45]. In 2005, Medicare Australia expenditure for ART services totalled $108.4 million, a 117% increase from 2003 [41].

While there has been a steady increase in the number of live births attributed to ART [39], the success of ART declines with advancing maternal age. Maternal age and type of infertility have been shown to be the two most important factors in predicting ART outcomes [46]. Medicare data showed that utilisation of ART is increasing at a greater rate in women over 42 years of age compared to women less than 42 years of age [41]. The proportion of Medicare claims for ART services made by women aged 35 to 44 years rose from 45% in 1997/98 to 61% in 2007/08 [47].

Women's reported experiences of ART include negative psychosocial [48,49] and physical outcomes [50] which are responsible for discontinuation of ART treatments among some couples [51]. ART use is also associated with increased maternal and perinatal morbidity [52,53], and health service utilisation [54]. In addition the cost implications of undertaking ART are significant [42].

Having children is often assumed to be a socially expected function of being a woman [55] and a defining aspect of femininity [56], providing identity and status to women [57]. For many women the experience of infertility may be dominated by feelings of grief, anger and humiliation [48]. Perceptions of self-stigma and reports of social censure have been identified among infertile women [58]. Paradoxically, while developments in ART have increased treatment options and therefore pregnancy as an outcome for some couples, some women report that they feel compelled to continue to seek medical intervention because of the social stigma attached to infertility and childlessness [59,60].

The pressure to conceive against a backdrop of declining fertility, together with the decreased potential of orthodox medical interventions, means that some women become 'desperate' to try anything to achieve parenthood. Despite literature showing more Australian women are using ART to achieve parenthood, and that world-wide women use CAM for reproductive health, only a limited number of studies were found reporting on women's CAM use to enhance fertility [61-66]. One recently published study reported a 30% lower pregnancy and live birth rate among concurrent users of CAM and ART compared to non-users of CAM [66].

There is now an increasing body of qualitative research literature on CAM use [27,28,30,35,67] including women's use of CAM for reproductive health [68] and experiences of infertility and ART [59,69-71], however no qualitative research was found specifically relating to women's use of CAM to enhance fertility. Among the studies reporting on CAM use to enhance fertility, the proportion of women or couples using CAM to enhance fertility or treat infertility varied considerably from 12% [61] to 91% [62]. This may be somewhat explained by the various definitions of CAM [61,63-66] and small sample sizes of many studies [62,65]. Most studies did not explore reasons for using CAM for fertility enhancement.

There have been calls for further research into women's use of CAM for reproductive health, both to provide evidence of safety and efficacy [72] and to ensure that clinicians are aware that a growing proportion of women who consult them may be using CAM [73]. The aim of this paper is to discuss the findings of focus groups conducted to explore the views and experiences of women who use CAM to enhance their fertility in Melbourne, Australia and the perspectives of the CAM practitioners they consult.

## Methods

Three focus groups were conducted in Melbourne, Australia between October and December 2007; two with women who used CAM and ART concurrently to enhance their fertility and one with CAM practitioners specialising in women's health.

Public health researchers have recently begun to utilise qualitative methods to explore peoples' feelings, values and experiences of life-changing events, including experiences of infertility [74], in an attempt to understand how and why people behave as they do [75,76]. We used focus groups to gain a preliminary understanding of the issues related to this little known area. Historically, focus groups have been used in reproductive health research especially among marginalised groups to 'give a voice' and empower participants [77]. Focus groups can stimulate discussion through group dynamics - the social interaction between and the relevance of the topic to the group members; however people may also feel uncomfortable about disclosing or discussing sensitive issues with strangers [78]. The study was approved by the Human Research Ethics Committee, La Trobe University (FHEC07/116).

### Recruitment

CAM practitioners and women using CAM to enhance their fertility were recruited from five CAM practices in metropolitan Melbourne that specialise in women's health. The decision to recruit women from CAM practices rather than ART clinics was made as little is known about why women choose to use CAM for fertility enhancement or their experiences of CAM and we wanted to specifically recruit women seeking CAM treatments. Flyers with study information and contact details were left in the participating CAM practices over three months (October to December 2007). Interested practitioners and women were instructed to contact the research team directly if they wanted to participate, to separate the recruitment of participants from the practitioner/client relationship.

### Participants

Eight CAM practitioners participated in the first focus group. They were all female practitioners with extensive clinical experience (mean time in clinical practice ten years, range three to 20 years) and most had multiple CAM qualifications. Their practice covered a range of modalities including naturopathy (n = 5), acupuncture (n = 3), western herbal medicine (n = 5), traditional Chinese medicine (n = 1), and psychology (n = 1).

Two focus groups, held with women who were using CAM to enhance their fertility, included a total of seven women. Although ten women initially expressed an interest in participating, reasons cited for non-attendance included other commitments including ART treatments and work responsibilities. While every effort was made to maintain women's privacy and confidentiality, at the first focus group, two women who attended were known to each other. Each was offered the opportunity of participating in a later focus group, however both were happy to stay and participate. At the conclusion of the focus group these women informed the researchers that knowing someone with similar experiences was reassuring.

Participating women had a median age of 40 years (range 34 to 44); one was currently pregnant and the remainder had not achieved parenthood. All had post-secondary education and private health insurance; three were single and four were partnered (three with male partners and one a female partner). Six women were using or had used CAM concurrently with ART. Three women had a medical diagnosis of infertility. One woman was utilising CAM modalities to enhance her fertility as preconception care. She was also consulting an ART specialist prior to using ART procedures to conceive as she did not have a male partner.

### Conduct and content of focus groups

The focus groups were held in different locations: two at different CAM practices in metropolitan Melbourne and the third at Mother and Child Health Research, La Trobe University, also in metropolitan Melbourne. Focus group guides were developed specifically for the study. Issues explored with both practitioners and women related to women's use of CAM to enhance fertility in conjunction with ART and to enhance fertility generally; their expectations of CAM and ART; and women's satisfaction with both the modalities used and the practitioners they consulted. Practitioners were also asked about referral practices, the modalities they use and their views on why women consult them. Written informed consent was obtained from each participant prior to the focus group discussion and the discussions were digitally recorded.

### Analysis

The three focus group discussions were transcribed verbatim with all references to individuals and organisations removed, then checked against the audio recordings for missing words or mistakes. The transcripts were thematically analysed by two of the investigators independently (JR, RC) then agreement reached regarding emergent themes. Thematic analysis was used, as exploratory studies require inductive identification of themes from the transcripts. A thematic conceptual network was used to connect basic codes into organising categories and finally global themes with quotes from the focus groups [79]. Data analysis proceeded with reading and re-reading the transcripts to ensure the transcripts were fully explored and guarantee emergence of basic codes. Basic codes were interrogated to ensure they were fully defined and to elicit organisational categories. This included identification of the frequency, intensity and extensiveness of the codes across all three focus group transcripts [78]. Final analysis of all organisational categories elucidated three global themes to form the basis of the analytical argument [80].

Quotes are used to illustrate the findings (the most frequent organisational categories which formed the three key themes) and are contextualised by the inclusion of the pseudonym given to women, as well as their age, and by the inclusion of CAM practitioners' mode of practice and their years of experience.

## Results

Three key themes around women's use of CAM for fertility enhancement emerged from the analysis of all the focus groups: *women's strong desire for motherh*ood; *women's negative experiences of ART; *and *women's positive experiences of CAM*.

### Women's strong desire for motherhood

The dominant theme to emerge from the focus groups was women's strong desire for motherhood. This desire was described by women as a willingness to try almost anything to maximise their chances of becoming pregnant, reflected in the variety of CAM modalities they invested in and their use of ART. Five women reported the unsuccessful use of ART to date (with one woman pregnant and one who had not started ART treatment), and all spoke of their desire to maximise their chances of achieving motherhood.

*I'm feeling desperate, absolutely desperate. I would try anything and everything to reach that goal. I'm very aware of my body, you know from that cervical mucus tracking, temperature tracking, all that sort of stuff and doing that every month and not conceiving and thinking I'm doing everything right, it really is excruciating. So yeah, I think desperation (Bernadette, 40yrs)*.

*I also have a view that I'll try anything no matter how kind of crazy it seems. Sometimes my husband just rolls his eyes when I say "I'm going to see a hypnotherapist or kinesiologist" or "I've snuck a couple of crystals into my handbag" or something like that. If someone suggested that I should sit in my back garden and howl at the moon I would do it (Carole, 42yrs)*.

CAM practitioners also spoke about women's overwhelming desire to achieve motherhood:

*There's the group that come who've done five, ten simulated cycles with no success, who are often stressed. They're coming to see what we do. "I've tried everything else, what is there?" (Acupuncturist and Birth Attendant, 6 years experience)*.

*Some of them come because they're desperate and they want to do everything, so they're at the stage where they just want to throw everything at it (Naturopath, 7 years experience)*.

### The stigma of infertility

Intrinsic to the desperation to achieve motherhood, was the stigma associated with infertility. While stigma was not specifically named, women spoke about the emotional difficulties they faced having to talk about their infertility to others; and the issue of having pregnant friends and/or family who seemed to have no difficulty in achieving a pregnancy, which was a constant reminder of their own inability to conceive.

My husband's friends were younger... it's just kind of like salt in the wound. I mean they're just falling pregnant. So it's very hard to talk to your own friends about, I just find it very hard because it's heartbreaking to actually have to talk about you know not being able to get pregnant when they just look at their husbands and that's it (Bernadette, 40yrs)

*The reaction from different girlfriends is really interesting. Some haven't asked very much at all and still seem to be quite, like they don't want to ask the question "Are you pregnant yet?" So they just don't bring it up and others have never asked. I think there's a little bit of a taboo about talking about infertility (Gai, 40yrs)*.

*People say helpfully "Maybe if you just go on holidays". My mother said that and I said "Oh if I go to Italy will my eggs be younger?" (Carole, 42yrs)*.

### Advancing age and declining fertility

CAM practitioners identified different groups of women who use CAM for fertility enhancement including: women who want a 'natural' experience; those who use ART but are worried about the side-effects, so draw on CAM to maintain their health and wellbeing; women who have had two or three cycles of ART and want to maximise their success in future cycles; and increasingly the most common group - women who are generally *older *with complex reproductive histories, have had numerous unsuccessful cycles of ART and who are exploring other options.

*Women come in for various reasons. Some want to get fit and healthy before they get pregnant and some have already got fertility problems. Others have been trying for over ten years. [They are] various ages but more the 30 and 40 year olds (Naturopath, 7 years experience)*.

*Frequently they have a difficult or complicated reproductive history (Herbalist and Naturopath, 20 years experience)*.

*I think 90% of the clients are coming in for fertility related issues; more than half of those are doing ART and most have quite a detailed sort of history of fertility related problems or history of IVF (Acupuncturist, 3 years experience)*.

*I suppose 70-80% of the people I see are women seeking assistance with fertility and increasingly so using ART. Less preconception and more of the older age group with more complicated histories (Acupuncturist and Naturopath, 8 years experience)*.

Advancing age was an issue also raised by women. Some women reflected on the link between their age and declining fertility, which served to increase their desperation to achieve motherhood:

*Having hit 40 and I'm feeling desperate, absolutely desperate, I would try anything and everything (Bernadette, 40yrs)*.

*I do all of those natural things, I go to a lot of different natural therapists and I think my age is the issue and IVF does nothing to make your eggs younger (Carole, 42yrs)*.

### The use of multiple modalities

Women reported using multiple and diverse CAM modalities in (six in conjunction with ART) to enhance their fertility and maximise their chances of becoming pregnant. Seventeen different modalities were reported including yoga, meditation, healings, reflexology, hypnotherapy, kinesiology, traditional Chinese medicine, and Brazilian herbs. The most common modalities used were acupuncture, naturopathy, herbal medicines and dietary supplements. Most women consulted a variety of practitioners, generally from the same CAM practice (e.g. they might see a naturopath and an acupuncturist from the one practice), and a few reported self medication with over-the-counter supplements or therapies obtained via the internet.

*I do masses of different therapies which I've kind of found along the way (Debra, 44yrs)*.

*I saw everyone at ' [name CAM practice]'. I go downstairs for both naturopath and acupuncture; I do the herbal medicines on the side (Anna, 34yrs)*.

Women also reported consulting different medical fertility specialists over time and of progressing through a range of available infertility treatments, however their experiences of ART were not always positive.

### Reduced to body parts: Women's experiences of ART

The second theme that emerged from the focus groups was that while appreciative of the advances in fertility medicine, women reported that their experiences of using ART for fertility enhancement were often negative. They felt that they were reduced to being a series of body parts, subjected to technical and dehumanising procedures. They described the treatments as 'gruelling', 'stressful', 'damaging', 'humiliating' and 'destructive'. They discussed feelings of isolation and they felt their interactions with orthodox medical personnel were often impersonal and sometimes distressing.

*[It feels like you're] Being lost in the world of statistics, blood tests, results, and negatives. Horrific terms that never leave your mind, like "Oh that blood test is so high you're postmenopausal" (Gai, 40yrs)*.

*The obstetrician was like 'Oh my God you're 40, send thee to an IVF doctor' and as a result I spent most of last year going through a kind of a hideous experience (Carole, 42yrs)*.

*I made an egg but it was what they called empty. The doctor said "I'll give you one more chance" and hung up. That triggered a lot of anxiety (Debra, 44yrs)*.

### Non-disclosure of CAM use

Women reported concerns about disclosing their CAM use to orthodox fertility specialists because they felt they would not be taken seriously; previous responses had been dismissive or at best ambivalent, and consultations were usually rushed.

*I was a vegetarian for years and my GP said "Go and eat some meat and you might get pregnant". I mentioned it to my IVF doctor who said "Diet has nothing to do with getting pregnant, women in Africa get pregnant". So I thought I'm not going to mention all these other therapies because he was completely dismissing. You're just a uterus basically, that's it, nothing else, it's just your ovaries and that's it' (Gai, 40yrs)*.

*Often with IVF doctors they actually don't want to have a conversation with you. They don't actually have time to have a conversation. They've got you slotted in and you can't. Also half the time they treat you like you're a moron (Carole, 42yrs)*.

On the other hand, one woman reported that it was her ART specialist who recommended that she try acupuncture after she had one unsuccessful ovulation stimulation cycle and egg pick-up; and another that her specialist was 'open' to her consulting a CAM practitioner although her general practitioner (GP) was ambivalent.

*I guess what prompted me to do it [CAM] was the IVF program. There was a monthly newsletter which had stories from other women and a number of them mentioned that they'd tried IVF with acupuncture. It seemed to make the difference so I thought 'Ok well let's give it a go' and also my specialist did suggest that I'd consider trying acupuncture when we started the 2nd stimulated cycle (Fran, 38yrs)*.

*I said [to ART specialist] "So if I just hang out with the girls up at ' [CAM Practice]' and they do all of that I'm happy with that, are you happy with that?" And I didn't feel that he was dismissive of it but I told my GP and she said "Yeah but I don't want to know anything about it" (Elise, 39yrs)*.

CAM practitioners experiences with and attitudes to orthodox medicine was also mixed. Most practitioners reported that they referred women to orthodox medical specialists and to other CAM practitioners as required. They reported a variety of responses from orthodox medical specialists, cautiously commenting that it was 'dependent on the individual specialist', with some difficult to work with.

*You do get a few GPs saying "I suggest you see a naturopath", maybe, if they're younger. So you do occasionally get them (Naturopath and Herbalist, 7 years experience)*.

*There are some people who are just out and out rude, and they're not a lot of fun to deal with (Herbalist and Naturopath, 20 years experience)*.

While some practitioners also received referrals *from *orthodox medical specialists and one reported a close professional association with a medical fertility specialist, the general consensus among the group was that ART specialists had little knowledge about CAM modalities despite increasing numbers of women using their services.

### The stigma of ART

Women reported that in addition to the stigma associated with infertility, there was stigma attached to pregnancies conceived through ART as they were not conceived naturally. This compounded their sense of failure and inadequacy and two women reported that they had difficulties in disclosing their use of ART to conceive to family and friends.

*My mum has said a couple of times "Oh what will I do if someone says to me was it IVF?" (Fran, 38yrs)*.

*I really struggle with that [disclosing the use of ART]. I'm happy to tell people say within five to ten years of my age, but I don't know that I would with someone my mother's generation (Gai, 40yrs)*.

Consequently the uptake and use of CAM modalities by women for fertility enhancement was generally considered to be more 'natural', therefore the women perceived CAM use as less stigmatising. There was a sense that CAM for fertility enhancement was 'legitimate' as there was no technological or 'unnatural' assistance. Women could also talk about using CAM without disclosing that it is for fertility enhancement.

*I do [tell people I am using [CAM] but not for fertility. People at work say "What's that strange tea you're drinking?" that sort of thing, and I very rarely have caffeine these days so they know I'm seeing a naturopath; but you know I would say "It's to do with my thyroid"' or I tend to be pretty private about it (Carole, 42yrs)*.

### Comfort and control: Women's experiences of CAM

The final theme to emerge was the positive and affirming experiences with CAM practitioners reported by women. These experiences were associated with: a sense of regaining control of their bodies; the healthy focus of CAM including individualised treatments regimes; and the nature of the relationship they formed with their CAM practitioners.

### Regaining control over one's body

Women described 'a whole of body approach', 'a beginning as opposed to an ending', 'making a difference to your psyche', 'a more general rather than specific effect', and 'feeling in control'.

*You feel [you're] not in control throughout the whole IVF process. You've handed your body over to someone else. Doing acupuncture was what I'd chosen to do and I felt some control as a result. I find it really empowering cooking up my herbs because I'm actually seeing what's going in (Debra, 44yrs)*.

It's so emotional, such a mess doing IVF and the toll it takes on your body. I'm doing meditation and I have healings done and now I'm just sort of quite calm about the whole thing (Anna, 34yrs)

*Actually, doing acupuncture was something additional that I'd chosen to do and I felt some control as a result of doing that (Fran, 38yrs)*.

### CAM as an individualised approach to fertility enhancement

It was also the individualised, health focus approach to fertility enhancement with CAM that many women also found appealing.

*Regeneration of your reproductive system, which alternative therapy does, talk about the possibilities for that, I think that's a really big difference for your psyche (Debra, 44yrs)*.

*I think the alternative therapies provide a great contribution, helping to counter balance that [negative effects of ART] (Gai, 40yrs)*.

Discussing the various treatment options for fertility enhancement, CAM practitioners emphasised that interventions were tailored for individual women, and involved a variety of CAM modalities and personal lifestyle modifications.

*From the naturopathic approach it is very individual, because everybody is so different; the issues they're coming in with, the individual's health problems. So we would use herbs, nutritional supplements, and flower essences (Naturopath, 7 years experience)*.

*For the person who is having 16 cans of coke and five coffees a day, I'd probably say "There's a lot of work we can do with just improving your overall health. I can identify a number of subclinical factors that may be affecting your overall health that may be affecting your fertility." To give them the sense that there are things they can do that are going to have some impact on overall health, and that may then make some change to fertility. I mean, that's very broad, but I would say "So I recommend you come and see one of our naturopaths and do some of that lifestyle stuff". I can do other supportive things and describe how the acupuncture would back that up (Acupuncturist and Birth Attendant, 6 years experience)*.

Some women had used CAM therapies previously and all felt CAM was 'safe' because it was 'natural'.

*I've been into natural health, complementary medicines, for 10 years anyway (Bernadette, 40yrs)*.

*I've been really familiar with Chinese medicine for a long time and found it more beneficial than western medicines for all sorts of things (Debra, 44yrs)*.

*Throughout my adult life I tended not to go for the drug solution if I could avoid it. I'm very much into preventative medicine and healthy lifestyle to try to deal with any health issues (Carole, 42yrs)*.

### Relationships with CAM practitioners

Although orthodox fertility treatments were also expensive, women felt using CAM was about choice, and about relationships based on trust and respect.

*I think it's the [medical] system to an extent. Perhaps complementary therapies are slightly outside that system and don't have those constraints and they [CAM practitioners] give you the opportunity to develop relationships a little bit more (Gai, 40yrs)*.

*I connect really well with her, and this process is one of the good things - having another woman to talk to. I didn't shop around at all on price. I was much more interested in the feeling I got from the place and their [practitioners] attitudes. Natural medicine is the most kind to yourself approach, and you can actually have conversations with the practitioners, as well as being treated (Carole, 42yrs)*.

Women reported that feeling healthy and having a good relationship with a healthcare provider was more important than cost; all were willing to pay the out-of-pocket expense for CAM use.

*I mean cost is important. But the relationship with the therapist is certainly part of it. I wouldn't be going regularly to see someone if I didn't have a relationship with them (Bernadette, 40yrs)*.

*The difference between going from that experience [ART] to going to the naturopath and sitting down and chatting with a lovely woman who talks about when I'm pregnant, is a radical shift in the way you perceive what you're doing and how you relate to your body. Profoundly different and that was a huge benefit, a blessing for me (Gai, 40yrs)*.

Some women also reported that they would continue to use CAM even if they did not have private health insurance and irrespective of any evidence of efficacy because of the value they placed on the relationships with practitioners and the control they gain from this choice.

## Discussion

This exploratory study provides some preliminary knowledge regarding women's and CAM providers' views and experiences of CAM use to enhance fertility. Consistent with the literature, women in our study expressed a strong desire to achieve motherhood, which they considered was part of their identity as women [55-57]; and reported feelings of stigma and humiliation associated with their inability to conceive [48] and their use of ART [60,81]. The women in this study were similar to Australian women using ART, i.e. they were older, had more education and had private health insurance [39]. They also used a variety of CAM modalities which they perceive to be natural, as has been reported by others [36,63,82] and did not disclose their CAM use to the fertility specialists they consult for fear of ridicule, which has also been reported in the literature [10,18,35,83].

CAM practitioners reported seeing an increased number of women for fertility enhancement who are also using ART, many of whom are older. This is consistent with the general trend of more Australians using CAM as a health care option [18,19]; the sociodemographic profile of Australian CAM users [18,19]; and the concurrent use of CAM in conjunction and orthodox medical regimes [10,18].

The use of CAM for fertility enhancement was prompted by the unsuccessful use of ART for all but one woman, who was using CAM as preconception care prior to using ART to conceive. Five women reported negative experiences of ART associated with short consultation times; a limited knowledge of CAM modalities among some orthodox medical specialists; and the mechanistic and disempowering affect of ART procedures. These reasons are consistent with the literature on CAM use especially in relations to dissatisfaction with or unsuccessful use of orthodox medicine [27,28]; the need for more control in decisions [22,29-34]; the treatment of chronic conditions [20,29,30,32]; the impersonal nature of orthodox medicine versus the naturalness of CAM [33,35]; and the nature of the interactions with CAM practitioners [33,36,37]. Women in this study were also desperate to achieve parenthood, related to the use of CAM for fertility enhancement reported elsewhere [65].

The unsatisfactory didactic style of communication from medical practitioners (ART specialists and GPs) reported by women in this study supports other findings [35,75]. Coulter and colleagues (1999) reported similar responses from focus group participants with chronic ailments, including infertility [75]. Participants valued a less paternalistic practitioner relationship, the provision of up-to-date information including different treatment options, and individual involvement in treatment decisions [75]. Vickers and colleagues (2006) reported women's past negative experiences with their GPs provoked their reluctance to disclosure CAM use [35].

The women participating in our study reported that they valued the information, support and sensitive communication provided by CAM practitioners, as well as the whole of person approach to fertility enhancement. The quality of the woman-practitioner relationship, especially communication style, and practitioners' acknowledgement of women as individuals, were important to women's overall experience of both ART and CAM. Sirois (2008) reports the motivation for CAM use in Canada has shifted away from 'push' factors such dissatisfaction with orthodox medicine to 'pull' factors including the holistic approach of CAM [31]. The women were satisfied with CAM and willing to pay out-of-pocket to access these services because practitioners provide them with some hope of achieving motherhood.

Our findings point to the importance of the client-practitioner relationship and its significance for women seeking fertility enhancement. Recent literature suggests that trust in one's health provider is integral in the face of uncertainty [84], and that trust is important in the clinical encounter [85]. For infertile women, the need for a trusting relationship may be heightened. They have experienced a loss of certainty in their body's fertility, and may also have experienced psychologically stressful medical encounters that do not result in guaranteed outcomes. The relationship with a CAM practitioner may therefore represent an integral way of regaining trust in one's body and the therapeutic encounter.

The limitations of this study include its small sample size, and the self-selected nature of the participants. It was based on the experiences of women who participated in three focus groups recruited from five CAM clinics that specialised in this area of care in Melbourne, Australia. Recruitment to focus groups is always difficult, even more so when the issue is of a sensitive nature [78]. CAM practices were chosen as the site for recruitment because the specific aim of the study was to understand why women use CAM to enhance fertility as little is known about this issue. Recruitment was undertaken over several months and despite a number of women expressing interest in participation, fewer women attended the focus groups than originally intended. Our intention had been to include women who were not using ART (women using CAM for preconception care or women only using CAM for fertility enhancement), however participation reflected what CAM practitioners reported - the majority of their fertility enhancement practice was with women also using ART. While we do not know what our findings may have been if the women participating were more diverse in their reasons for using CAM, the findings about motivations for CAM use reflect those reported in the literature on CAM use more generally, i.e. dissatisfaction with or poor outcomes associated with orthodox medicine [27,28]; a need for more control [22,29-34]; the impersonal nature of orthodox medicine [33,35]; and the personalised nature of the interaction with CAM practitioners [33,36,37].

## Conclusions

Strategies to enhance communication between women, fertility specialists and CAM practitioners in the provision of fertility services need to be developed. Further research is needed to explore the experiences of CAM use; the benefits or harms of combining ART and CAM to enhance fertility, as well as more qualitative work around women's experiences of ART and CAM and why women choose or do not choose to use CAM for fertility enhancement.

## Competing interests

The authors declare that they have no competing interests.

## Authors' contributions

JR conceived the study; all authors contributed to the design of the study; JR, HMcL, and RC conducted the focus groups; and JR and RC analysed the data. All authors contributed to the manuscript and read and approved the final version

## Pre-publication history

The pre-publication history for this paper can be accessed here:

http://www.biomedcentral.com/1472-6882/9/52/prepub
